# Global Workflow of a Comanipulation‐Based Robotic System for Cervical Spine Surgery

**DOI:** 10.1002/rcs.70193

**Published:** 2026-06-13

**Authors:** A. Koszulinski, J. Sandoval, C.‐T. Wu, S.‐H. Huang, S. K. Sahani, S.‐L. Wu, T. Essomba, T. Vendeuvre, J.‐P. Faure, S. Zeghloul, M. A. Laribi

**Affiliations:** ^1^ Department of GMSC Pprime Institute CNRS ENSMA University of Poitiers UPR 3346 Poitiers France; ^2^ Nantes University École Centrale Nantes CNRS LS2N UMR 6004 Nantes France; ^3^ Department of Neurosurgery Chang Gung Memorial Hospital at Linkou Chang Gung University Taoyuan Taiwan; ^4^ Graduate Institute of Biomedical Electronics & Bioinformatics National Taiwan University Taipei Taiwan; ^5^ Department of Computer Science and Information Engineering Chang Gung University Taoyuan Taiwan; ^6^ National Central University Taoyuan City Taiwan; ^7^ ABS Laboratory College of Medicine and Pharmacy University of Poitiers Poitiers France

## Abstract

**Background:**

Cervical arthrodesis requires precise pedicle screw placement to ensure safety and effectiveness. Traditional planning and execution are time‐consuming and prone to variability.

**Methods:**

We developed a robot‐assisted system integrating three components: an AI‐based preoperative planning module, adapted from previous work, to generate patient‐specific screw trajectory from 3D CT point‐clouds; an intraoperative registration and motion compensation system with optical tracking to align the trajectory with patient anatomy in real time; and a comanipulation control strategy enforcing virtual fixtures and depth limits to guide the robotic arm safely. The system was tested on 3D‐printed models and cadaveric specimens.

**Results:**

Robotic assistance significantly improved the geometric accuracy of drilling, reducing transverse positional deviations by a factor of two and orientation deviations by a factor of eight compared with freehand procedures. In addition, the average drilling depth overshoot was reduced by 50%. Perforation rates were found to be of the same order as those observed with freehand techniques.

**Conclusion:**

The proposed workflow improves trajectory‐following accuracy and depth control while preserving intuitive surgeon interaction. These results demonstrate the feasibility of integrating AI‐based planning, intraoperative tracking, and collaborative robotics for cervical spine surgery.

## 1 Introduction

Cervical arthrodesis is a fundamental surgical strategy for the treatment of a wide spectrum of cervical spine pathologies, including degenerative, traumatic, neoplastic, and inflammatory conditions. The primary objective of this procedure is to achieve spinal stability through the fusion of adjacent vertebrae using implant‐based fixation constructs.

Accessing the vertebrae for implant fixation can be done through anterior (front) or posterior (back) approaches, chosen based on pathology, lesion extent, and surgical team preferences [1]. Various fixation techniques exist, differing in the anatomical anchorage for the spinal implant screw(s) required to stabilise the spine [2]. The precise screw placement requires detailed anatomical knowledge of the vertebrae, including entry point location, trajectory orientation, diameter, and insertion depth. During the surgery, the accurate positioning of the surgical drill along the planned trajectory is critical to ensure both mechanical stability and patient safety [1].

**FIGURE 1:**
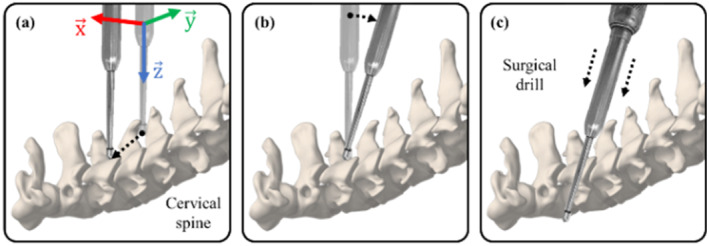
Three stages of the pedicle drilling process: (a) tool positioning via translational movements, (b) orientation adjustment with rotations around $\vec{x}$ and $\vec{y}$ axes, (c) drilling along the $\vec{z}$‐axis while maintaining alignment.

The manipulation of the surgical drill towards the pedicle sight can be divided into three stages, each characterised by different degrees of tool mobility. In the first stage, the tip of the surgical drill is positioned at the desired drilling entry point, primarily requiring three degrees of translational mobility. Once the drill tip is correctly placed, the second stage involves adjusting its orientation to align the drill axis with the planned drilling trajectory. This step is crucial to prevent vertebral perforation and ensure the integrity of surrounding structures. This requires controlling rotations around the tool's $\vec{x}$ and $\vec{y}$ axes, as illustrated in Figure 1. The tool's self‐rotation around its longitudinal axis ($\vec{z}$) does not influence the drilling direction. Finally, the third stage consists of drilling along the longitudinal axis ($\vec{z}$). Throughout this step, the tool must remain aligned with the preselected trajectory, compensating for patient motion, particularly that induced by breathing.

Integrating robotic systems into the operating room enhances surgical precision and repeatability, enabling finer drilling and improved screw stability.

In conventional freehand spine surgery, the literature reports pedicle screw misplacement rates ranging from 8.3% to 50.3% [3], highlighting the technical difficulty of accurate screw insertion. The introduction of navigation systems has significantly reduced these positioning errors, with reported rates of perfectly placed screws ranging from 89% to 100% in navigated procedures [4].

More recently, robotic‐assisted systems have demonstrated further improvements in placement accuracy, particularly for pedicle screws in the thoracic spine, where pedicles are smaller and surgical precision is even more critical [5]. Beyond accuracy, robotic integration in spine surgery has also been associated with reduced revision rates, lower infection rates, shorter operative times, and faster patient recovery, as reported in large clinical cohorts [6]. In orthopaedic surgery, robot‐assisted procedures now account for 56% of pedicle screw implantations, followed by total hip and knee arthroplasties [7].

Robotic surgical systems can be categorised into three types. First, autonomous robots fully execute procedures without direct surgeon control, such as the TSolution One (formerly Robodoc) by THINK Surgical (USA), developed for total hip arthroplasty [8]. Second, teleoperated systems are controlled remotely through a master‐remote interface; although primarily developed for soft‐tissue surgery, exploratory trials have been conducted in orthopaedic contexts using platforms such as the Da Vinci system [9]. Third, comanipulated robots, currently the most prevalent in orthopaedic surgery, assist the surgeon by combining human expertise with robotic precision [10]. To enhance collaboration, the robot can utilise a multi‐scale graph convolutional neural network with temporal attention, as proposed in [11]. Real‐time analysis of the surgeon's skeletal movements yields an action‐recognition accuracy of 94.16%, allowing for context‐appropriate robotic responses. Practical experiments demonstrate that this approach improves both the fluidity and safety of surgeon‐robot interaction.

Within the comanipulated robots category, two main control paradigms can be identified: (1) *guidance‐based systems*, in which the robotic arm acts as a trajectory guide, as exemplified by ROSA Spine (Medtech, France) [12], Mazor X Stealth (Medtronic, USA) [13], and ExcelsiusGPS (Globus Medical, USA) [14]; and (2) *direct manipulation systems*, in which the robotic arm is rigidly coupled to the surgical instrument and physically follows the surgeon's movements, while virtual constraints enforce adherence to the planned trajectory, as implemented in platforms such as VELYS (J&J Medtech, USA) and MAKO (Stryker, USA) for knee and hip arthroplasty [15].

The primary advantage of direct manipulation lies in preserving the surgeon's control over the procedure. Continuous comanipulation enables a collaborative interaction that combines the surgeon's dexterity and clinical expertise with the positioning accuracy and motion‐constraining capabilities of the robotic device, thereby enhancing precision while maintaining intuitive control.

However, despite these advantages, currently available robotic systems for spine surgery are exclusively based on the guidance‐based paradigm. To the best of our knowledge, no robotic system dedicated to spine surgery implements a true direct‐manipulation approach with axis‐constrained collaborative drilling. This limitation motivates the present work.

Building upon this gap, this paper introduces a novel robotic assistance platform for spine surgery based on direct comanipulation. The main contributions of this work are threefold. First, we integrate an AI‐based preoperative planning module, adapted from our previously reported cervical screw trajectory planning framework [16], into the proposed robotic workflow. This module automatically determines optimal pedicle drilling trajectories from 3D vertebral data, thereby reducing planning time and limiting inter‐operator variability compared with commercially available systems relying on manual trajectory definition. Second, we developed a vertebra registration and motion compensation strategy that maintains alignment between the planned trajectory and the patient anatomy throughout the procedure. Third, we implemented an admittance‐based comanipulation control scheme that constrains tool motion to the drilling axis while allowing intuitive surgeon guidance and depth limitation through virtual fixtures. Therefore, the principal contribution of this study is the system‐level integration of these components into a coherent workflow linking preoperative planning, intraoperative alignment, motion‐aware robotic positioning, and surgeon‐guided constrained drilling.

The remainder of the paper is organised as follows. Section 2 presents the global workflow and system overview. Section 3 describes the AI‐based preoperative planning method, Section 4 details vertebra registration and intraoperative motion compensation, and Section 5 introduces the comanipulation control strategy for pedicle drilling. Section 6 reports experimental validation on 3D‐printed and cadaveric specimens, and Section 7 concludes the paper.

## 2 Global Workflow and System Overview

**FIGURE 2:**
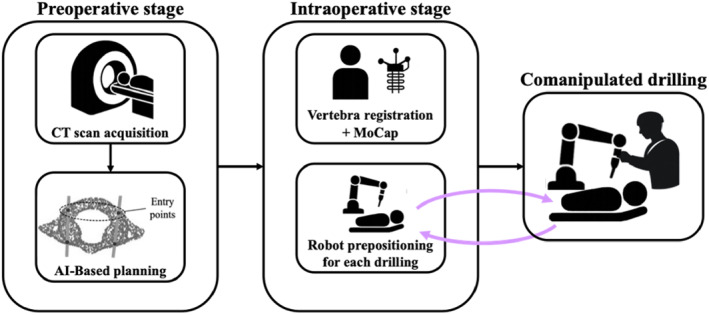
Workflow of robot‐assisted cervical arthrodesis, including preoperative trajectory planning, intraoperative vertebra registration with robot prepositioning, and comanipulated pedicle drilling with virtual constraints.

The proposed system follows a three‐stage workflow that combines AI‐based preoperative planning, intraoperative vertebra registration with motion compensation, and robot‐assisted comanipulated drilling. The complete process, illustrated in Figure 2, is designed to ensure accurate trajectory selection, reliable alignment between the patient and the robot, and safe human‐guided drilling.

### 2.1 Preoperative Stage: CT‐Based Planning of Drilling Trajectories

**FIGURE 3:**
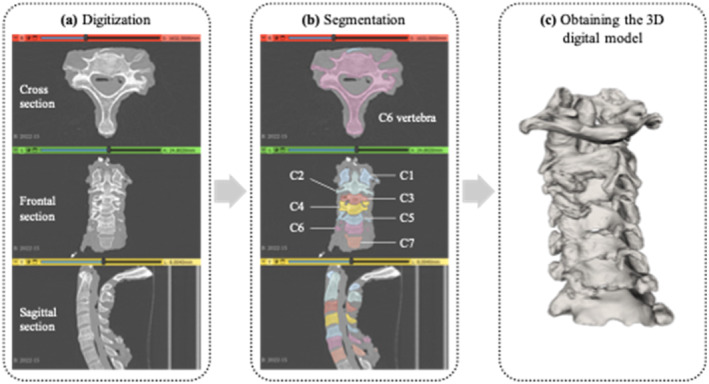
Steps involved in creating the 3D digital model of the cervical spine, including (a) CT imaging of the anatomical specimen, (b) segmentation of the vertebrae, and (c) reconstruction of the final digital model.

The workflow begins with the acquisition of a CT scan of the patient's cervical spine. Using this medical imaging technique, a 3D digital model of each vertebra is reconstructed through segmentation of the anatomical structures of interest (Figure 3a–c). This step isolates the relevant bone regions required for trajectory computation. It is important to note that this digital model constitutes the geometric foundation for the planned surgical act.

Once the digital model is available, optimal drilling trajectories are automatically computed using a deep learning‐based planning framework (Section 3). The algorithm identifies suitable entry points, orientation axes, screw diameters, and drilling depth limits based on vertebral surface features. Compared to commercial systems in which surgeons manually define and refine trajectories, this automatic approach significantly reduces preoperative planning time and standardises screw placement.

### 2.2 Intraoperative Stage: Vertebra Registration and Robot Positioning

During surgery, the planned trajectories must be transferred from the digital model to the patient's anatomy. To achieve this, CT‐to‐patient registration is performed using an optical motion capture system capable of tracking both the vertebrae and the robot platform. This registration maintains consistency between the planned trajectories and the patient's actual anatomy despite intraoperative motions such as breathing or small manipulations by the surgical team.

Following registration, the robot is automatically prepositioned in a pose that places the surgical drill close to the selected entry point, with an orientation approximating the corresponding planned trajectory. This prepositioning reduces the physical effort required by the surgeon and prepares the system for comanipulated drilling. The registration framework and motion compensation strategies are described in detail in Section 4.

### 2.3 Comanipulated Drilling and Sequential Execution

Once properly positioned, the surgeon performs drilling using a comanipulation strategy in which the robotic arm is physically guided while enforcing virtual fixtures. These constraints restrict the drill tip to remain aligned with the planned axis and prevent approaching unsafe anatomical regions. Depth‐limiting constraints further ensure protection of critical structures, such as the vertebral artery and spinal cord.

After completion of a drilling task, the robot automatically repositions itself for the next trajectory, returning to the prepositioning step of the intraoperative stage. This closed‐loop process enables consecutive screw placements while maintaining precision and safety throughout the procedure. The control strategy that ensures safe and intuitive comanipulated drilling is presented in Section 5.

## 3 AI‐Based Preoperative Planning

The automatic preoperative trajectory planning module adopted in this study is adapted from the patient‐specific screw planning framework proposed by Chang et al. [16], which leverages 3D deep learning on vertebral point‐cloud representations, and is integrated into the proposed robotic workflow. This approach was originally developed for posterior C1–C2 cervical fixation and is particularly suited to anatomically complex regions where manual trajectory definition is time‐consuming and highly operator‐dependent. In the present study, this planning module is used as the upstream trajectory‐generation component of the robotic workflow. While its quantitative validation was originally performed for C1–C2 fixation, the workflow demonstrates how AI‐generated trajectory descriptors can be linked to registration, robot positioning, and comanipulated execution in a broader cervical surgery setting.

A dataset consisted of 50 paired C1–C2 CT‐derived vertebral models from Chang Gung Memorial Hospital. Ten cases were reserved for independent testing, and the remaining 40 cases were used for training after data augmentation. Augmentation by flipping, resizing, rotation, and translation generated 11,520 datasets, which were split into 9126 training and 2304 validation point‐cloud datasets. Ideal screw entry and exit points were initially annotated on 3D models by experienced technicians and subsequently validated by neurosurgeons. These points were converted into surface‐based sub‐cloud clusters, with 60 points per cluster selected after optimisation, because this cluster size provided the best balance between segmentation accuracy and screw‐path recovery.

### 3.1 Point‐Cloud Representation of Vertebral Anatomy

Following CT acquisition and segmentation, each cervical vertebra is converted into a three‐dimensional point cloud model representing the external bone surface. Point clouds provide an efficient and flexible description of complex vertebral geometry while remaining well suited for deep learning architectures designed to process unordered spatial data [17].

For each vertebra, a fixed number of surface points is uniformly sampled from the segmented model to form the network input. This representation preserves the global morphology and local geometric features necessary for identifying anatomically valid screw trajectories, while maintaining robustness to minor segmentation variations.

The network was based on the PointNet architecture and included a T‐net module for input alignment, three feature‐transformation stages using convolution, batch normalisation, and ReLU activation, global max pooling for feature aggregation, fusion of local and global features, and final fully connected layers for point‐wise semantic segmentation. The model was implemented in TensorFlow 2.6 and trained on an NVIDIA RTX 3090 GPU for 200 epochs, with a learning rate of 0.001 and a batch size of 32; the total training time was approximately 12.6 h.

### 3.2 Learning‐Based Identification of Entry and Exit Region

**FIGURE 4:**
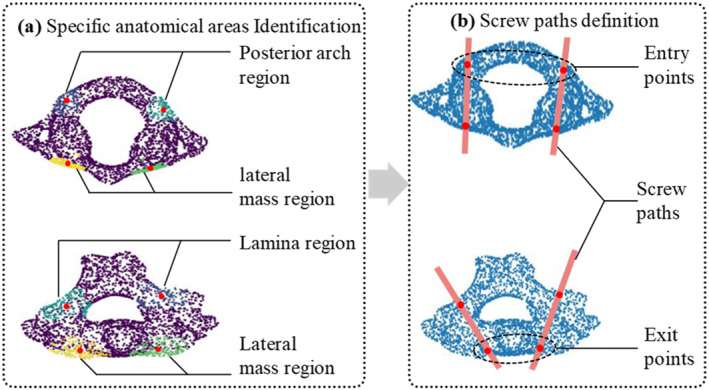
Overview of screw trajectory planning formulated as a 3D point‐cloud semantic segmentation problem. Expert‐defined entry and exit locations are represented as surface clusters (a), and the final drilling trajectory is reconstructed from the predicted clusters (b).

Rather than directly regressing a drilling axis, the method expresses screw planning as a 3D semantic segmentation problem. As illustrated in Figure 4, the annotated entry and exit landmarks are represented as 60‐point surface clusters, allowing each screw anchoring site to be learnt as a labelled region on the point cloud.

Two dedicated PointNet‐based segmentation networks were trained for C1 and C2, respectively. Each network predicts point‐wise labels for the entry and exit regions, enabling the system to identify clinically acceptable screw anchoring zones from patient‐specific vertebral geometry. Model performance for entry/exit region segmentation was evaluated using mean Intersection‐over‐Union (mIoU), while the clinical relevance of the predicted regions was assessed through trajectory recovery metrics, including path deviation, point deviation, surface proximity, and surgeon‐based Gertzbein grading. During inference, the trained network segments the input point cloud into anatomical regions corresponding to the predicted entry and exit clusters. This strategy allows the model to learn entry and exit regions from patient‐specific vertebral morphology while preserving clinically defined trajectory constraints.

### 3.3 Trajectory Reconstruction From Segmented Clusters

Once the entry and exit clusters are identified, the geometric centre of each cluster is computed. Because the vertebral model consists solely of surface points, the final entry and exit locations are defined as the surface points closest to these cluster centres. Connecting the resulting entry and exit point yields the final drilling trajectory.

This process constrains each trajectory to the patient‐specific vertebral surface while preserving compatibility with posterior cervical fixation principles and local variations in pedicle size, laminar orientation, and curvature. The mean path deviation values were 0.88 mm, 0.70 mm, 0.95 mm, and 0.83 mm for left‐entry, left‐exit, right‐entry, and right‐exit points, respectively. The corresponding mean point deviation values were 2.05 mm, 1.31 mm, 2.32 mm, and 1.30 mm. The minimum surface proximity across all test cases was 1.04 mm, with mean values of 1.75 mm on the left and 1.61 mm on the right. In addition, experienced neurosurgeons assessed the predicted trajectories using the Gertzbein grading system, and all predicted paths were graded as clinically acceptable, indicating no relevant cortical breach.

### 3.4 Planning Outputs for Robotic Execution

For each planned trajectory, the algorithm outputs the three‐dimensional entry point, exit point, drilling‐axis orientation vector, and planned depth. These parameters form the geometric trajectory descriptor required for intraoperative registration, robot prepositioning, and comanipulated execution. Within the proposed system, the planning module functions as a planning‐to‐robot interface, transforming patient‐specific vertebral geometry into trajectory descriptors that can be registered, tracked, and enforced by the robotic control system.

It should be noted that the quantitative validation of this AI planning module was originally performed for C1–C2 fixation. In the present study, the module is integrated as a planning‐to‐robot interface within a global robotic workflow. Full AI‐based planning validation for subaxial cervical levels C3–C7 remains a future work and will require dedicated level‐specific annotation, training, and testing because these vertebrae differ from C1–C2 in morphology, screw corridor geometry, and fixation strategy.

## 4 Vertebra Registration, Patient Motion Compensation and Robot Positioning

### 4.1 Dry (3D‐Printed) and Wet (Cadaveric) Models' Preparation

Since each vertebra in the digital cervical spine model is initially independent, the vertebrae are first fused (rigidly connected) by adding material between adjacent vertebral bodies, corresponding to the intervertebral discs. The resulting cervical spine is then attached to a base that allows stable placement on a flat surface. To transfer the planned drilling trajectories from the digital model to the physical model, a marker cluster is fixed to the same base. This cluster corresponds to the one used on the patient during navigated surgery and enables the motion capture system to localise the spine in space and establish the correspondence between the physical model and its digital counterpart.

### 4.2 Automatic Robot Alignment

Drilling operations are performed without direct visualisation of the planned trajectory, meaning that the surgeon relies exclusively on the virtual fixtures generated by the robotic system. To ensure that drilling trajectories follow their planned axes, an automatic robotic alignment procedure is integrated into the control architecture.

**FIGURE 5:**
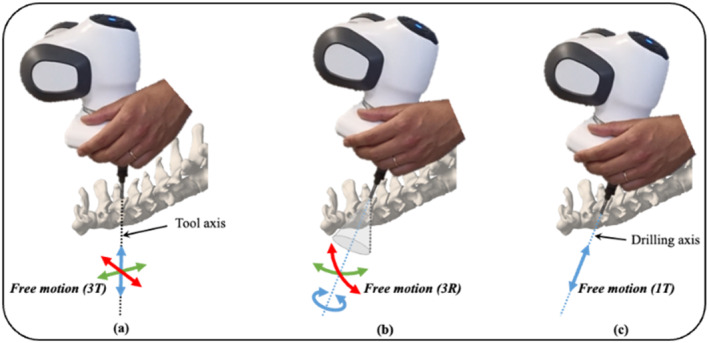
Steps for using the robotic assistance platform in the surgical context: (a) free positioning and (b) alignment of the tool along the desired drilling axis, and (c) drilling execution, where the tool's free movements are constrained to motion strictly along the planned drilling axis.

The alignment process consists of three steps. First, the robot end‐effector moves along the vertical axis while maintaining its orientation, positioning the drill tip on a horizontal plane approximately 10 cm above the spine (Figure 5a). This initial motion creates a safe clearance to prevent any unintended contact with the anatomy. In the second step, the tool moves across this horizontal plane to position the drill tip on the planned axis, 5 cm from the drilling entry point. During this motion, the tool inclination is also adjusted to align its axis with the planned trajectory (Figure 5b). The tool orientation around $\vec{x}$ and $\vec{y}$ axes allows matching with the planned drilling trajectory, whereas the rotation around the $\vec{z}$‐axis (the tool's longitudinal axis) is used to avoid singularities and to facilitate comfortable handling by the surgeon. Finally, in the third step, the robot constrains its motion to allow the practitioner to move the drilling tool exclusively along the planned drilling axis (Figure 5c).

## 5 Comanipulation Control for Pedicle Drilling

This section presents the control strategy enabling safe, surgeon‐guided pedicle drilling while enforcing virtual constraints derived from the pre‐planned trajectory. The approach relies on an admittance‐based control strategy to allow intuitive physical interaction during tool positioning, while ensuring axis‐constrained motion and depth limitation during drilling.

### 5.1 Control Architecture

**FIGURE 6:**
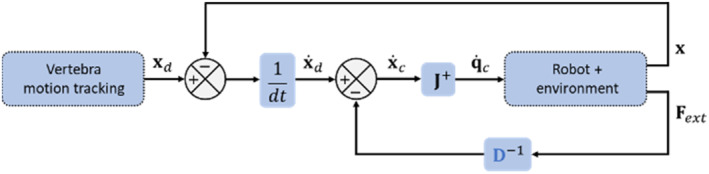
Admittance‐based control architecture enables co‐manipulated robotic assistance during pedicle drilling.

To enable comanipulation, an admittance‐based control strategy is implemented on the robotic platform, namely a 7‐DoF Franka Emika manipulator. While previous approaches have relied on impedance control [18], admittance control provides a comparatively stiffer dynamic response, thereby better ensuring adherence to the predefined drilling trajectory. Figure 6 presents the control architecture adopted in this study.

In this framework, the robot is velocity‐controlled at the joint level. The desired Cartesian motion is computed from the relative displacement of the vertebra with respect to the robot, as measured by the motion capture (MoCap) system. This formulation enables active compensation of vertebral motion while preserving intuitive surgeon–robot interaction.

The admittance law is implemented using the inverse of a diagonal damping matrix D, which maps the estimated external forces $F_{\text{ext}}$ applied at the surgical drill into cartesian velocity commands.

The external forces ($F_{\text{ext}}$) are estimated via the robot's internal joint torque sensors, which are calibrated and processed through a low‐pass filter to reject high‐frequency noise while preserving the tactile feedback essential for surgical guidance. Furthermore, the frame transformations, specifically the mapping from the tool's end‐effector frame to the base frame of the manipulator, and have provided a comprehensive description of the signal conditioning pipeline, including the specific calibration protocols used to ensure the accuracy of the force‐to‐velocity admittance law during the drilling interaction.

The joint velocity control signal is defined as,

(1) $${\dot{q}}_{c}=J^{+}{\dot{x}}_{c}=J^{+}\left({\dot{x}}_{d}-D^{-1}F_{\text{ext}}\right),$$

where $J^{+}$ denotes the Moore‐Penrose pseudo‐inverse of the robot Jacobian matrix. To explicitly express the variables in the end‐effector frame {E}, Equation (1) is rewritten as,

(2) q˙c=J+x˙d−XEODE−1FextE,

where XEO is the spatial transformation matrix between the base frame {O} and the end‐effector frame {E}. The diagonal matrix $D_{E}^{-1}$ allows tuning the influence of the external forces along each translational and rotational direction of the end‐effector.

Defining the force‐induced Cartesian velocity as,

(3) $${\dot{x}}_{F_{E}}=D_{E}^{-1}F_{ext_{E}}.$$

Equation (2) becomes,

(4) q˙c=J+x˙d−XEOx˙FE,

During the drilling stage, the damping matrix is configured such that motion is allowed only along the longitudinal drilling axis. The resulting Cartesian velocity contribution becomes,

(5) $${\dot{x}}_{F_{E}}={\left[\begin{array}{cccccc} 0 & 0 & \frac{F_{\text{ex}t_{E_{z}}}}{{D_{E}}_{z}} & 0 & 0 & 0 \end{array}\right]}^{T}$$

which enforces rigid behaviour in all directions except translation along the $\vec{z}$‐axis, corresponding to the drilling direction.

### 5.2 Evaluation of Motion Compensation

A comparative study was conducted to evaluate the robot's performance of the motion compensation based on the control law used: an admittance‐based approach. The robot's performance was assessed during a motion tracking task. This experiment aimed to evaluate the accuracy with which the robotic device could track the movements of a cluster of markers representing the patient.

**FIGURE 7:**
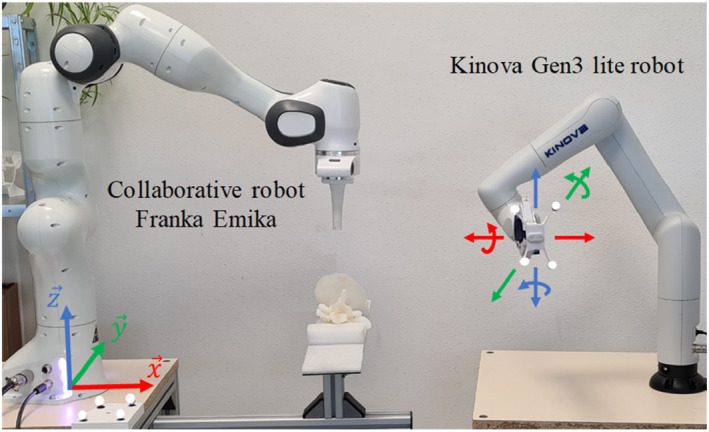
Initial setup of Franka Emika and Kinova Gen3 lite robots.

To generate an identical target trajectory during successive evaluations of the robot controlled by admittance, a second robotic device was used: the Kinova Gen3 lite robot, at the end of which a cluster of markers was positioned (Figure 7). The movements of this cluster were recorded in real time by the Optitrack motion capture system and sent as commands to the robot via its controller.

The movements performed by the Kinova robot consisted of three translational movements followed by three rotational movements. Given that during a real operation, the movements perceived by the patient are primarily due to their breathing, the Kinova robot's translational movements were generated at a speed similar to that of rib cage movement during respiration, averaging 10 mm/s [19].

**FIGURE 8:**
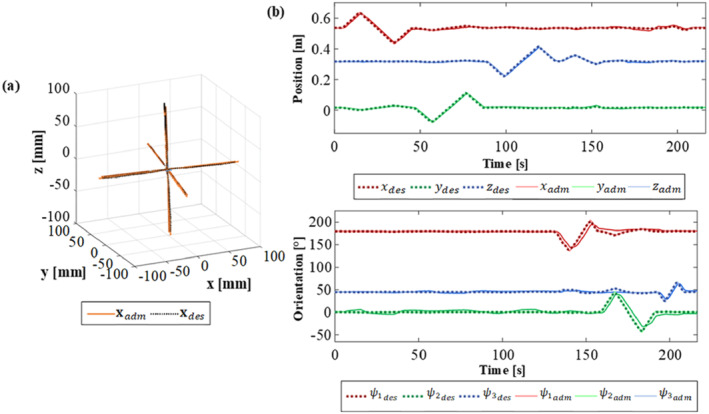
(a) Trajectory followed by the robot's admittance‐controlled end effector relative to the desired trajectory represented in the initial end effector frame and (b) evolution of the position and orientation of the robot's end effector during the execution of the tracking task relative to the desired trajectory represented in the robot coordinate system.

Throughout the test, the evolution of the position and orientation of the end effector of the Franka Emika robot, as well as that of the placement of the marker cluster attached to the end of the Kinova robot, were recorded (Figure 8).

**FIGURE 9:**
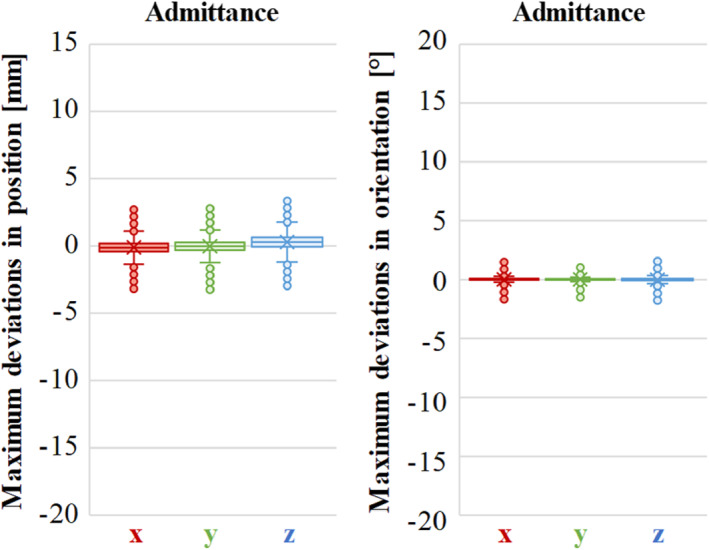
Maximum deviations in position and orientation measured during trajectory tracking for admittance control.

The position of the cobot's end effector was then compared to the target trajectory defined from the movements observed at the marker cluster during the trajectory‐following task. Figure 9 then shows the deviations of the cobot from the desired tracking trajectory.

According to the results obtained using admittance control, the maximum deviation along the *z*‐axis is 3.3 mm. Regarding the cobot's tracking of orientation changes in the marker cluster, the maximum deviation along the *z*‐axis is 1.8°.

### 5.3 Compliance Modulation for Axis‐Constrained Drilling

Once the tool is positioned and aligned with the planned trajectory, the drilling assistance mode is activated. In this mode:

- The robot exhibits rigid behaviour in all directions transverse to the drilling axis, ensuring that the tool remains aligned with the planned access path;
- The surgeon retains direct control of the longitudinal motion of the tool along the $\vec{z}$‐axis, benefiting from compliant behaviour that preserves tactile sensations comparable to conventional drilling;
- The MoCap system continuously tracks vertebral motion, and the robot actively updates its Cartesian reference to compensate for these displacements, thereby maintaining alignment with the planned drilling trajectory throughout the procedure.

**FIGURE 10:**
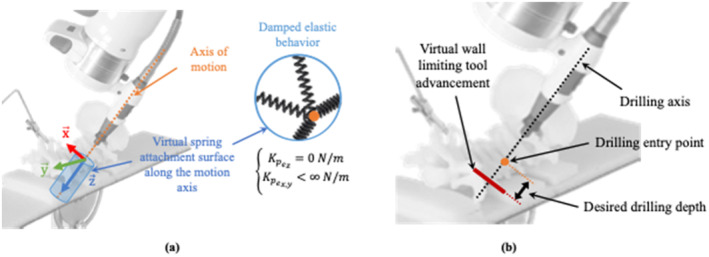
Admittance‐based comanipulation strategy for pedicle drilling: (a) axis‐constrained motion enforced by virtual fixtures aligned with the planned drilling trajectory, and (b) depth limitation generated by a virtual wall at the desired drilling depth.

Axis‐constrained drilling is achieved by assigning very high stiffness, or equivalent, high damping in the admittance formulation, in the five constrained directions (three translations and two rotations), while maintaining a low stiffness, or equivalently a low damping value, along the drilling axis. This configuration allows free advancement along the $\vec{z}$‐axis while preventing lateral of angular deviations that could result in cortical breach. Figure 10 illustrates this strategy, where virtual fixtures restrict motion to the drilling axis and ensure safe comanipulation.

### 5.4 Compliance Modulation for Depth Limitation

To prevent excessive penetration of the drill and to protect critical structures located anterior to the vertebral structure, a virtual depth‐limiting constraint is implemented. The objective is to allow smooth drilling over most of the trajectory while progressively increasing opposition as the tool approaches the desired depth.

**FIGURE 11:**
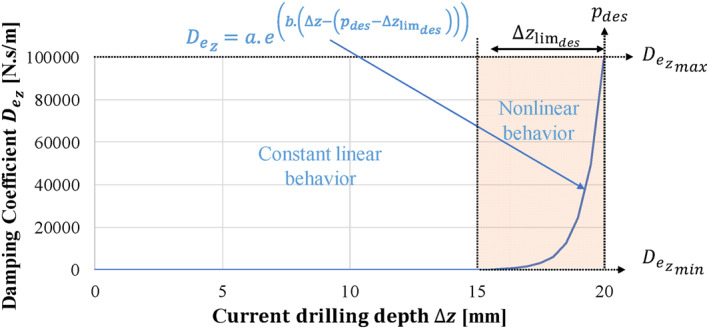
Nonlinear damping (stiffness) profile along the drilling axis progressively increases as the tool approaches the desired drilling depth to generate a smooth virtual wall.

This behaviour is achieved by defining a nonlinear evolution of the damping coefficient ${D_{e}}_{z}$ along the drilling axis: For shallow depths, $D_{e_{z}}$ remains at its minimum value $D_{e_{z_{\min}}}$, ensuring a compliant response similar to freehand drilling. Once the tool reaches a predefined depth threshold, the damping increases progressively according to an exponential law:

(6) $${D_{e}}_{z}=\left\{\begin{array}{c} a\;\exp\left(b.\left(\Delta z-\left(p_{\text{des}}-\Delta z_{\lim_{\text{des}}\;}\right)\right)\right),\text{if}\;\Delta z\geq p_{\text{des}}-\Delta z_{\lim_{\text{des}}\;}\;\\ {{D_{e}}_{z}}_{\min},\text{if}\;\Delta z\;<p_{\text{des}}-\Delta z_{\lim_{\text{des}}\;}\; \end{array}\right.,$$

where Δz is the current drilling depth, $p_{\text{des}}$ the desired drilling depth, and $\Delta z_{\lim_{\text{des}}\;}$ a depth margin (set to 5 mm) over which the nonlinear damping transition is applied. The exponential parameters a and b are selected so that the damping reaches ${{D_{e}}_{z}}_{\min}$ (set to 100 N.s/m) exactly at the desired depth, while ensuring a continuous and smooth evolution of the damping profile, thereby avoiding abrupt variations in the commanded velocity that could lead to oscillatory behaviour. The exponential parameters are calculated as,

(7) $$a=\frac{{{D_{e}}_{z}}_{\max}}{\exp\left(b.p_{\text{des}}\right)}.$$

and,

(8) $$b=\frac{\ln\left(\frac{{{D_{e}}_{z}}_{\max}}{{{D_{e}}_{z}}_{\min}}\right)}{\Delta z_{\lim_{\text{des}}\;}},$$

where ${{D_{e}}_{z}}_{\max}$ is set to 100,000 N.s/m and $p_{\text{des}}$ to 20 mm. This nonlinear modulation results in a virtual wall that gently opposes the surgeon's motion as the tool approaches the target depth, ultimately preventing overruns while preserving natural haptic sensations. Figure 11 shows the resulting damping evolution profile.

From a control perspective, the proposed nonlinear damping modulation is designed to ensure a stable and physically consistent interaction during human‐robot coupling. The damping matrix remains diagonal and strictly positive, which guarantees a passive behaviour of the admittance model under bounded interaction forces. The continuity of the exponential profile further avoids abrupt variations in velocity, thereby preventing oscillatory responses near the depth limit.

In practice, the parameters of the damping law are experimentally tuned to ensure smooth and stable behaviour during drilling. Moreover, all experiments were conducted in regions of the robot workspace sufficiently far from kinematic singularities, ensuring a well‐conditioned Jacobian and stable computation of the pseudoinverse. While a formal analysis of passivity, including the effects of discretisation, saturation, and sensor noise, is beyond the scope of this study, these aspects are considered as part of ongoing work.

## 6 Experimental Validation

Following the development of the platform control, various experimental tests were carried out with the participation of surgeons.

All the procedures performed in studies involving human cadavers complied with the ethical standards of the institutional research committee and with the requirements of the current version of the Declaration of Helsinki.

**FIGURE 12:**
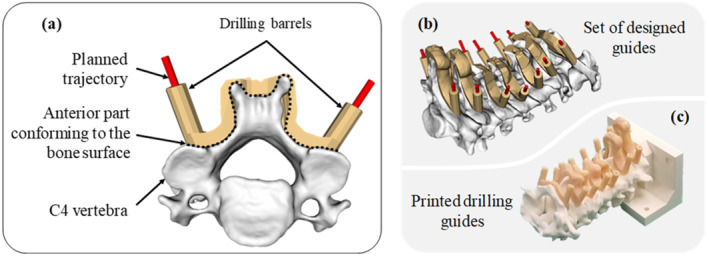
(a) Design of the drilling guide for vertebra C4 with an anterior portion conforming to the shape of the vertebral bone surface and a posterior portion consisting of the drilling barrels associated with the planned drilling trajectories, (b) overview of the drilling guides designed on the digital model for vertebrae C1 to C7 of the anatomical spine and (c) printed drilling guides.

This section first presents the drilling tests conducted at the Pprime Institute (University of Poitiers) on printed models of the cervical spine (Dry model). These tests required the integration of a procedure for automatically aligning the robotic device with the planned drilling directions. Subsequently, assisted drilling tests were conducted at the ABS Lab, the Anatomy, Biomechanics, and Simulation Laboratory at the University of Poitiers, on a donated spine extracted from a human cadaveric specimen (wet model). For this study, drilling guides (Figure 12) were used to locate the planned drilling trajectories on each vertebra of the anatomical spine. This is a standard localisation procedure used in the operating room [20, 21].

The registration strategy used in the experimental setup differs from clinical workflows. The following items have to be emphasised:

- Marker‐based registration in this study may overestimate registration stability compared to real surgical conditions, where factors such as soft tissue movement, segmental motion, and line‐of‐sight interruptions can introduce additional errors.
- Clinical robotic spine surgery commonly employs bone‐fixed reference frames or intraoperative CT‐based registration, which provide higher robustness and are less susceptible to soft tissue artefacts.
- Therefore, the current results should be interpreted as a proof‐of‐concept validation of the integrated robotic workflow, rather than a direct representation of clinical accuracy.

### 6.1 Dry Model (3D‐Printed) Experiments

The experiments were conducted on printed models corresponding to the replica of an anatomical cervical spine, obtained through medical images for exactitude in geometry and dimension. Several steps were necessary to prepare the desired tests.

After defining the desired drilling paths, the cervical spine model was printed in several copies for surgeons to perform the drilling. Before this printing step could be carried out, the digital model of the cervical spine was first modified.

**FIGURE 13:**
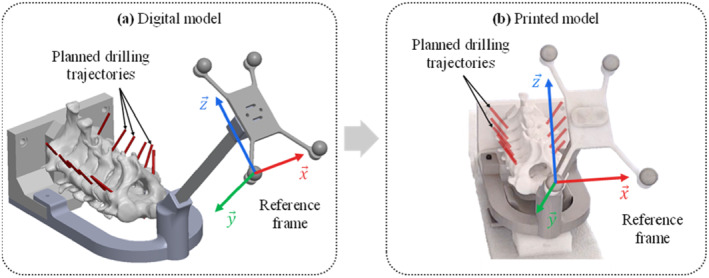
Reference frame definition associated with (a) the digital model and (b) the printed model.

In order to be able to identify the drilling trajectories planned on the digital model on a printed model, a cluster of markers was fixed on the same base as the model (Figure 13a). This cluster of markers corresponds to that associated with the patient during navigated surgery (Figure 13b).

The results obtained following the drilling tests carried out on the printed Dry model are presented from two points of view: on the one hand from the point of view of roboticists, by analysing the precision of the gestures carried out by surgeons with robotic assistance, and on the other hand, from the point of view of surgeons, by evaluating the number of drillings correctly carried out according to a clinical criterion.

The performance of the robotic assistance platform was first compared to the surgeons' skill by evaluating the deviations measured between the drill and the desired drilling trajectory for each test carried out ‘freehand’ and then with robotic assistance. This first comparison allows evaluating the contribution of the robotic assistance platform to the precision of the drilling gestures carried out by the surgeons.

**FIGURE 14:**
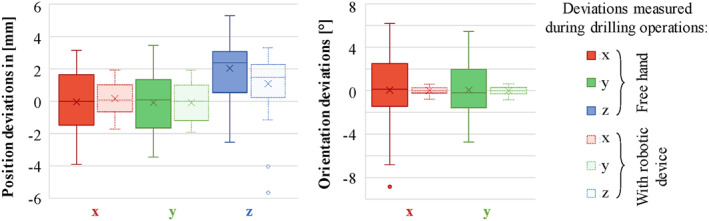
Maximum measured deviations between the tool and the desired drilling paths for drilling performed with and without robotic assistance on the dry models.

The data were measured in reference frames associated with each drilling, built from the tool reference frame when the drilling gesture was initialised, i.e. when the drill was aligned with the desired drilling trajectory. The $\vec{z}$‐axis, corresponding to the longitudinal axis of the tool, thus represents the drilling axis. The maximum deviations, positive and negative, from the desired drilling paths were recorded for each drilling. Figure 14 thus represents the maximum deviations recorded for the 38 drillings carried out with the robotic device and the 25 drillings carried out ‘freehand’.

**FIGURE 15:**
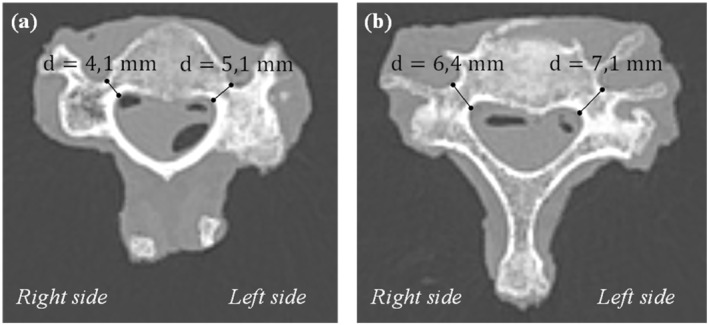
Measurement of pedicle diameters on transverse sections of vertebrae (a) C3 and (b) C6 using 3D Slicer software.

The results obtained show deviations in the transversal directions to the drilling axis that are half as significant in position and eight times lower in orientation when drilling is carried out with robotic assistance compared to those carried out ‘freehand’ (see Figure 15). Furthermore, the desired drilling depth limit is exceeded on average by 4 mm for drilling carried out “freehand”, and by 2 mm for drilling carried out with the robotic device. These observations highlight a gain in precision offered by the robot for maintaining a straight trajectory when drilling.

A second assessment was then conducted to determine whether the drillings were clinically performed correctly. Specifically, the criterion considered was the occurrence of perforation of the vertebral bone surface. A specific grade can be assigned based on the severity of the perforation [22].

**FIGURE 16:**
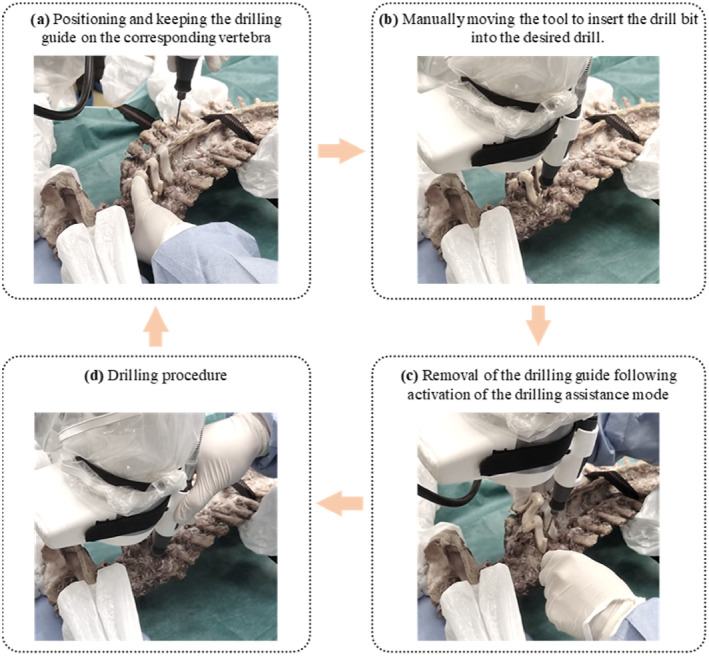
Steps were followed when performing robotic‐assisted drilling on the anatomical spine.

The number of perforations observed on the printed models following drilling performed with and without robotic assistance was evaluated based on the minimum diameter of the pedicles of each vertebra. These diameters were estimated using 3D Slicer software (Figure 16), on transverse sections of the cervical spine scan, in which the pedicle diameter was the smallest (Table 1). The obtained data are presented in Table 2.

**TABLE 1:** The diameter of the vertebral pedicles.

Cervical vertebra		C2	C3	C4	C5	C6	C7
Pedicle diameters [mm]	Left	6.8	5.1	5.5	6.4	7.1	7.3
	Right	6.6	4.1	5.2	6.2	6.4	8.1

**TABLE 2:** Number of cortical perforations recorded after drilling performed with and without robotic assistance.

Cervical vertebra	Number of perforations recorded for drilling done ‘freehand’		Number of perforations recorded for drillings performed with the robotic device	
	Left	Right	Left	Right
C2	0	0	0	1 (I)
C3	1 (I)	2 (I)	0	3 (I)
C4	0	1 (I)	0	1 (I)
C5	0	0	0	0
C6	0	0	0	1 (L) + 1 (I)
C7	1 (I)	0	1 (*I*)	1 (I)
Total	5 perforations/25 drillings (20%)		9 perforations/38 drillings (24%)	

Abbreviations: I, inferior perforation; L, lateral perforation.

### 6.2 Wet Model (Cadaver) Experiments

Drilling tests were conducted on the anatomical cervical spine from which the digital model was obtained in the previous section. As in previous experiments, drilling was performed with and without robotic assistance. The surgeon performed half of the drilling using the robotic assistance platform (on one side), and the other half with a hand drill using 3D‐printed drilling guides to guide his movements (on the other side). The objective of this second study is to compare the effectiveness of the virtual walls generated by the robotic device. A drilling guide was defined for each cervical vertebra of the anatomical spine studied from the digital model [21].

**FIGURE 17:**
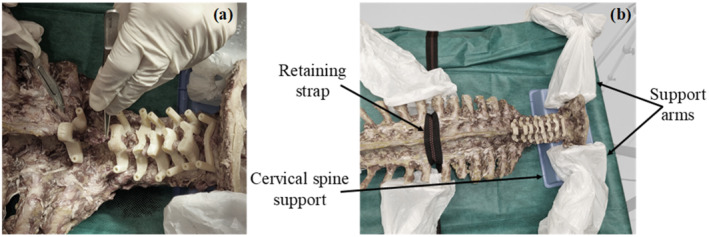
(a) Dissection of the anatomical spine performed by the surgeon to remove residual muscle tissue and allow positioning of the drilling guides and (b) immobilisation of the anatomical spine on the operating table.

The surgeon dissected the spine to remove residual muscle tissue from the posterior surfaces of the vertebrae in preparation for positioning the drilling guides (Figure 12). This step was performed with particular care to preserve the ligaments between each vertebra (Figure 17).

Seven drillings were performed by the surgeon on the left side of the anatomical spine from vertebrae C1 to C7 with robotic assistance. For each drilling, a guide was first positioned on the target vertebra to localise the entry point (Figure 16a). The surgeon then manually guided the robot end‐effector to insert the drill tip into the guide barrel until contact with the bone surface was established (Figure 16b), ensuring accurate positioning at the planned entry point and alignment with the drilling axis.

Once the tool was correctly positioned, the robotic drilling assistance mode was activated. At this stage, the tool motion was constrained by the robot along the predefined drilling axis. The drill guide was then removed (Figure 16c), and the drilling was performed exclusively under robotic guidance (Figure 17d).

This protocol ensures that the guide is used only for initial localisation, while the drilling phase itself relies solely on the virtual fixtures generated by the robotic system.

**FIGURE 18:**
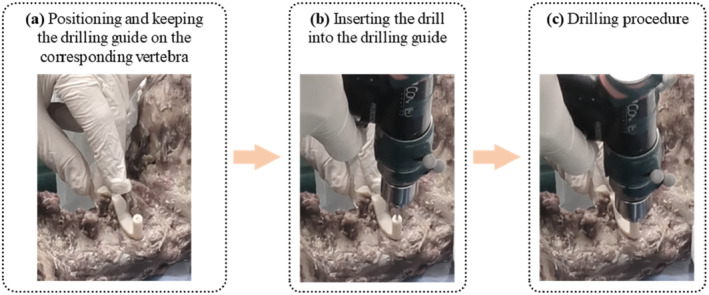
Steps followed when drilling with guides on the anatomical spine.

Seven other drillings were performed by the surgeon on the right side of the anatomical spine without robotic assistance. For these tests, the surgeon's actions were constrained only by the drilling guides then held on the vertebrae throughout the drilling (Figure 18).

**FIGURE 19:**
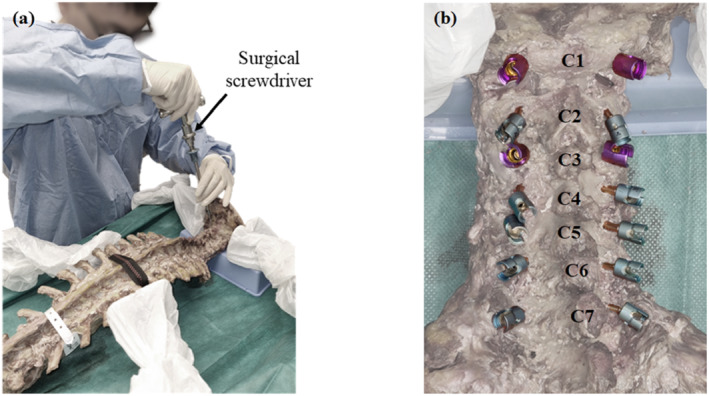
(a) Insertion of a screw into the bone canal made on the left side of the C1 vertebra and (b) set of inserted screws.

After all, the drilling was completed, the surgeon inserted 3.5 mm diameter screws into each cervical vertebra (Figure 19).

**FIGURE 20:**
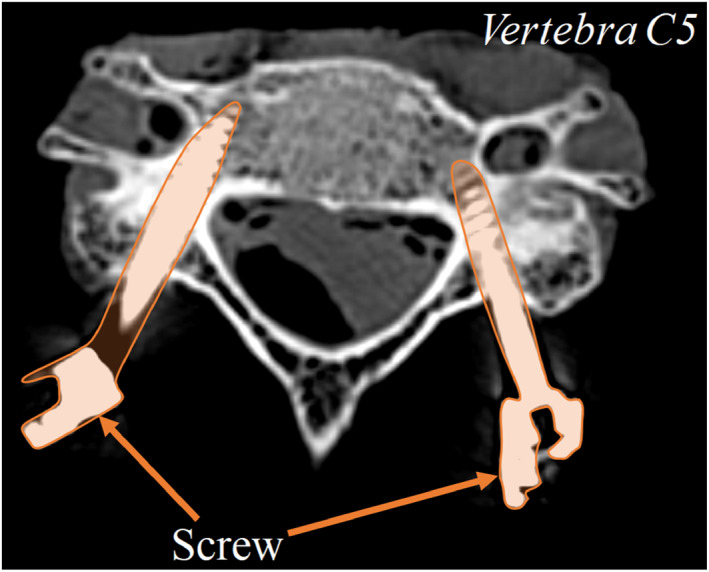
Cross‐sectional view of the screws inserted into the C5 vertebra.

Finally, a 3D scan of the anatomical spine was performed with an O‐arm to check for the presence of potential perforations within the cervical vertebrae (Figure 20).

### 6.3 Quantitative Evaluation

From a clinical perspective, each inserted screw can be assessed by assigning a perforation grade based on the extent to which the screw exceeds the bone surface of the vertebra in question. As shown Section 6.1, these perforations can be lateral, medial, inferior, and/or superior depending on their location relative to the vertebra.

Table 3 presents the perforation grades assigned to each screw by the surgeon who performed the experiments, according to the classification established by Sciubba et al. [20]. A total of three grade 2 perforations were recorded for drilling performed with guides, compared to two grade 2 perforations and one grade 1 perforation for drilling performed with robotic assistance.

**TABLE 3:** Perforation grades assigned to virtual screws reconstructed along the drilled channels.

Cervical vertebra	Perforation grade of the screws inserted following the drillings performed	
	Without robotic assistance	With robotic assistance
C1	0	0
C2	0	0
C3	2‐M/I	2‐I
C4	0	0
C5	0	0
C6	2‐M	1‐M
C7	2‐M	2‐M/I

Abbreviations: M, medial perforation; I, inferior perforation.

These results indicate that the severity of perforations obtained using robotic assistance remains comparable to that observed with guide‐assisted drilling. Combined with the geometric accuracy improvements reported in Section 6.1, this suggests that the proposed robotic system can achieve a similar level of clinical safety while providing enhanced control over trajectory and depth.

## 7 Conclusion

This paper presented an integrated workflow for robot‐assisted cervical arthrodesis that combines AI‐based preoperative trajectory planning, intraoperative vertebra registration with motion compensation, and an admittance‐based comanipulation strategy to support safe pedicle drilling. Together, these modules establish a coherent framework that maintains accurate alignment between planned and executed trajectories while preserving intuitive surgeon–robot interaction and enforcing depth‐limiting virtual constraints during drilling.

Experiments on dry (3D‐printed) models showed that robotic assistance reduced transverse positional deviations by approximately a factor of two and orientation deviations by nearly a factor of eight compared with freehand drilling, while also reducing the average depth overshoot from 4 to 2 mm. Although perforation rates remained of the same order as freehand procedures, these results demonstrate a clear improvement in the geometric fidelity of the drilling trajectories and highlight the importance of integrating anatomical constraints into the control strategy.

Wet (cadaveric) experiments confirmed the compatibility of the proposed workflow with standard surgical instruments and 3D‐printed drilling guides. The virtual constraints generated by the robot enabled control of tool motion along the drilling axis and contributed to limiting severe cortical breaches, with results comparable to guide‐assisted drilling. These findings suggest that comanipulation‐based robotic assistance can complement existing navigation and guide techniques by providing active axis and depth control while maintaining surgeon authority over tool advancement.

Overall, this study demonstrates the feasibility of a unified global workflow that integrates an AI‐based patient‐specific screw trajectory planning module with vertebra tracking and collaborative robotic execution for cervical spine surgery. Future work will focus on extending and validating the AI planning module for C3–C7, refining compliance modulation to further reduce perforation rates, and integrating additional sensing or real‐time imaging to enhance robustness and support clinical translation.

## Ethics Statement

All the procedures performed in studies involving human cadavers complied with the ethical standards of the institutional research committee and with the requirements of the current version of the Declaration of Helsinki.

## Conflicts of Interest

The authors declare no conflicts of interest.

## Data Availability

The data that support the findings of this study are available from the corresponding author upon reasonable request.
